# Genomic and evolutionary characterization of newly emerged highly pathogenic avian influenza H5N1 clade (2023–2025)

**DOI:** 10.14202/vetworld.2025.3745-3760

**Published:** 2025-12-10

**Authors:** Eman Abd El-Menum Shosha, Mohamed Khames Mohamd, Mostafa Abd Elmotiliub Shehata, Mahmoud Hashem Mohamed, Ibrahim Mohamed Eldaghayes, Mohamed Shaker Abdelhafez

**Affiliations:** 1 Department of Virology, Faculty of Veterinary Medicine, New Valley University, Kharga, Egypt; 2 Department of Avian and Rabbit Medicine, Faculty of Veterinary Medicine, New Valley University, Kharga, Egypt; 3 Department of Avian and Rabbit Medicine, Faculty of Veterinary Medicine, Assiut University, Asyut, Egypt; 4 Department of Aquatic Animal Medicine, Faculty of Veterinary Medicine, New Valley University, Kharga, Egypt; 5 Department of Microbiology and Parasitology, Faculty of Veterinary Medicine, University of Tripoli, Tripoli, Libya

**Keywords:** Clade 2.3.4.4b, genetic diversity, highly pathogenic avian influenza virus H5N1, One Health, phylogenetic analysis, Upper Egypt, vaccine mismatch

## Abstract

**Background and Aim:**

Highly pathogenic avian influenza virus (HPAI) H5N1 continues to threaten poultry biosecurity worldwide due to rapid antigenic drift and reassortment. Since late 2020, clade 2.3.4.4b strains have dominated outbreaks across multiple continents. This study genetically characterized H5N1 isolates circulating in Upper Egypt during 2023–2025, clarified their phylogenetic origin, and compared them with vaccine strains used nationally.

**Materials and Methods:**

A total of 100 samples from 25 broiler flocks showing respiratory and neurological symptoms across New Valley, Assiut, and El-Minya governorates were examined. Specimens were screened for avian influenza subtypes (H5N1, H9N2, H5N8, H6N2) and differential viral pathogens (Newcastle disease virus, infectious bronchitis virus, infectious laryngotracheitis virus, infectious bursal disease virus) using reverse-transcription quantitative polymerase chain reaction (RT-qPCR). Positive isolates were propagated in specific-pathogen-free embryonated chicken eggs and identified through hemagglutination and hemagglutination inhibition assays. Partial hemagglutinin gene sequencing and phylogenetic analyses were performed using Molecular Evolutionary Genetics Analysis version 7.0.

**Results:**

HPAI-H5N1 was detected in 16% (4/25) of flocks, showing 25%–50% mortality. Five isolates displayed high hemagglutination titers (7–8 log2) and were confirmed as H5N1 subtype by RT-qPCR. Phylogenetic analysis classified New Valley-1-H5N1-2023 and New Valley-2-H5N1-2024 within clade 2.3.4.4b. These strains shared 96%–99% nucleotide and amino acid identity with recent Egyptian and Eurasian H5N1 isolates but only 72%–84% with currently used Egyptian vaccine seeds (e.g., MEFLUVAC [Kemin Industries, Inc., USA], EgyFlu [Nagy Awad Group, Cairo, Egypt]). Mutations R72S, A83D, and T140A were identified in receptor-binding and antigenic regions of hemagglutination, implying potential antigenic drift.

**Conclusion:**

This is the first documentation of clade 2.3.4.4b HPAI-H5N1 circulation in broiler flocks of Upper Egypt. The low genetic relatedness to existing vaccine strains indicates probable vaccine mismatch and reduced protection. Continuous molecular surveillance, integration of full-genome sequencing, and periodic vaccine seed updates are essential for effective containment. Enhanced monitoring at the domestic–wild bird interface will help mitigate cross-species transmission and align with One Health strategies for zoonotic risk reduction.

## INTRODUCTION

Over the past decade, poultry and animal health have been increasingly challenged by a variety of viral infections, many of which are fatal. Among these, RNA viruses have been responsible for most major outbreaks due to their high mutation and recombination rates. Influenza viruses, in particular, exhibit the highest genetic variability among RNA viruses, driving continuous viral evolution and emergence of new pathogenic strains [1–4].

Avian influenza viruses (AIVs) are major pathogens that pose a persistent threat to global poultry production and public health. Belonging to the family Orthomyxoviridae, AIVs infect a broad range of avian and mammalian species. Their single-stranded, negative-sense RNA genome consists of eight segments encoding at least 11 proteins, including hemagglutinin (HA), neuraminidase (NA), matrix proteins (M1 and M2), nucleoprotein, polymerase subunits (PB2, PB1, and PA), and non-structural proteins (NS1 and NS2). Based on the antigenic diversity of HA and NA surface glycoproteins, AIVs are classified into 16 HA and 9 NA subtypes, identified across various avian species [5–8].

Wild migratory birds serve as the natural reservoirs for all AIV subtypes and play a central role in their global dissemination to domestic poultry populations [9, 10]. In domestic birds, AIVs are generally classified as either low-pathogenic or highly pathogenic (HPAI), with H5 and H7 subtypes responsible for severe disease and high mortality [11]. Since the 1990s, HPAI H5 strains have undergone substantial genetic evolution, driven by mutations and reassortment events, resulting in the formation of 10 phylogenetic clades (0–9) and numerous subclades based on HA gene characteristics [12, 13].

The first widespread transboundary dissemination of HPAI-H5N1 clade 2.2.1 occurred in 2005, after which it became endemic in several poultry populations [14]. A subsequent wave occurred in 2014, when HPAI-H5N8 clade 2.3.4.4a triggered extensive outbreaks across Europe, followed by the emergence of clade 2.3.4.4b, which spread from Asia to Europe and Africa during 2016–2017, leading to major poultry losses worldwide [15–17].

By 2020, diverse genotypes of HPAI-H5N1 belonging to clade 2.3.4.4b had emerged in wild birds and rapidly expanded across Africa, Asia, Europe, and North America [18–22]. During this evolutionary process, the *HA* gene of HPAI-H5N8 viruses within clade 2.3.4.4b reassorted with multiple low-pathogenic AIVs, generating a novel H5N1 subtype. This new lineage subsequently gave rise to at least 15 H5N1 genotypes and additional reassortant variants, such as H5N2, H5N3, H5N4, and H5N5 [12, 23]. Currently, HPAI-H5N1 clade 2.3.4.4b is the predominant subtype responsible for widespread outbreaks globally, affecting both wild and domestic birds and even infecting mammals. Notably, human cases were reported in the United Kingdom and Spain in 2022 [24, 25].

In Egypt, HPAI-H5N1 of clade 2.2.1 was first detected in 2006 and has persisted endemically in poultry for over a decade [26]. Between 2016 and 2018, HPAI-H5N8 viruses from clade 2.3.4.4b were also identified among migratory birds traversing major flyways, including the Black Sea–Mediterranean and East African–West Asian routes [27, 28]. Subsequent detection of HPAI-H5N8 strains in domestic flocks across multiple Egyptian governorates underscored their economic and epidemiological significance [29, 30]. Over time, the endemic H5N1 clade 2.2.1.2 was largely replaced by HPAI-H5N8 of clade 2.3.4.4b, making H5 the dominant subtype in Egypt’s poultry industry [31]. Alarmingly, Egypt continues to record the highest number of human H5N1 infections globally, along with cases of H9N2 infection [32]. In response, nationwide H5/H9 vaccination programs were initiated, aiming to maintain genetic and antigenic compatibility with circulating strains, especially within the commercial poultry sector [26].

Despite extensive global surveillance of HPAI H5 viruses, genomic information on the recently emerged H5N1 clade 2.3.4.4b strains in Egypt, particularly in Upper Egypt, is scarce and fragmented. Most previous Egyptian studies have focused on the Nile Delta and Lower Egypt, where dense poultry production facilitates the persistence of AIV. In contrast, the southern governorates (New Valley, Assiut, El-Minya) remain largely neglected in active molecular monitoring programs, even though they lie along major migratory bird corridors (Black Sea–Mediterranean and East African–West Asian flyways).

Furthermore, current vaccine seed strains used in Egypt (e.g., MEFLUVAC [Kemin Industries, Inc., USA], EgyFlu [Nagy Awad Group, Cairo, Egypt], Nobilis H5N2 [MSD Animal Health, New Jersey, USA]) were developed from earlier clades 2.2.1.1 and 2.2.1.2. These vaccines may no longer confer adequate protection against novel field variants that have undergone antigenic drift and reassortment within clade 2.3.4.4b. Limited molecular evidence is available on how far these new field isolates diverge genetically and antigenically from the vaccine strains currently in use.

In addition, the evolutionary linkages between the newly emerged Egyptian isolates and international H5N1 strains circulating in Europe, Asia, and Africa during 2021–2022 have not been clearly established. No published data so far document HPAI-H5N1 detection in broiler flocks of Upper Egypt, or assess their genetic mutations in receptor-binding and immunogenic epitopes (such as R72S, A83D, T140A) that could alter virulence and vaccine efficacy. This lack of localized molecular and evolutionary insight represents a significant research and surveillance gap for both veterinary and zoonotic preparedness.

This study aimed to isolate, identify, and genetically characterize newly emerged HPAI-H5N1 clade 2.3.4.4b strains currently circulating in broiler flocks of Upper Egypt (2023–2025). Specifically, the objectives were to:

1. Detect and confirm the presence of HPAI-H5N1 in field samples using hemagglutination/hemagglutination inhibition (HI) assays and subtype-specific reverse-transcription quantitative polymerase chain reaction (RT-qPCR) screening while ruling out co-infection with other major avian pathogens (Newcastle disease virus [NDV], infectious bronchitis virus [IBV], infectious laryngotracheitis virus [ILT], infectious bursal disease virus [IBDV]).
2. Sequence and analyze the partial *HA* gene to determine the phylogenetic position of the Egyptian isolates within the global H5N1 clade 2.3.4.4b lineage.
3. Assess genetic divergence and amino acid substitutions in antigenic and receptor-binding regions compared with contemporary vaccine strains and international reference viruses.
4. Evaluate evolutionary relationships and potential transmission routes linking Egyptian isolates to strains from neighboring continents, highlighting potential migratory bird or trade-related introduction pathways.
5. Generate genomic evidence to inform vaccine update strategies and national surveillance policies, contributing to the sustainable control of avian influenza and alignment with *One Health* and Sustainable Development Goal (SDG) 3 (Good Health and Well-Being) objectives.

## MATERIALS AND METHODS

### Ethical approval

All animal handling and sampling procedures in this study were conducted in compliance with institutional ethical standards and approved by the New Valley Research Ethics Committee, Faculty of Veterinary Medicine, New Valley University, Egypt (Approval No. 04-2024-100313). Informed consent was obtained from all poultry owners before sample collection. Tissue samples were obtained only from freshly dead birds. Tracheal and cloacal swabs were collected under strict biosafety precautions, including the use of laboratory coats, gloves, shoe covers, and post-procedure handwashing with soap followed by alcohol sterilization. All laboratory procedures involving HPAI-H5N1 virus isolation were performed in a biosafety level 3 facility.

### Study period and location

**Figure 1:**
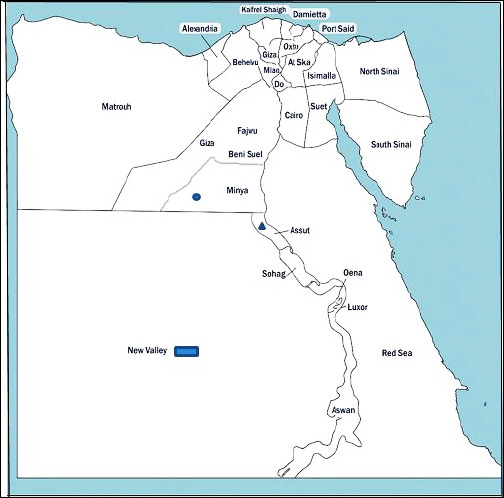
Distribution of highly pathogenic avian influenza virus-H5N1 of clade 2.3.4.4b in Upper Egypt governorates. El-Minya, Assiut, and New Valley governorates are represented by blue circles, blue triangles, and blue rectangles, respectively. The prevalence rate was 8% in New Valley Governorate and 4% in El-Minya and Assiut Governorates.

Between December 2023 and January 2025, a total of 100 clinical samples were collected from 25 broiler flocks located in different localities across the New Valley, Assiut, and El-Minya governorates in Upper Egypt (Figure 1). These areas were selected based on reports of respiratory distress, increased mortality, and suspected avian influenza outbreaks in commercial poultry farms.

### Sampling and specimen preparation (Table 1)

**Table 1:** Descriptive epidemiological data of flocks examined for avian influenza virus detection in the Upper Egypt governorates (New Valley, Assiut, and El-Minya).

Sampling governorate	Samples number	Year	Type and age of flock	Flock size	Mortality %	Collected samples	Vaccination programs	Prevalence rate (%)	Postmortem signs and lesions
**New Valley**	40	2023–2025	Broilers, 20–35 days	4000–9000	25–50	Tracheal swab; Cloacal swab; Pancreas; Lung; Trachea; Kidney; Brain	Inactivated H5N1 vaccine + some unvaccinated	8	Severe respiratory manifestations, neurological impairment, pulmonary congestion and edema, congested intestine, congested lung, general septicemia, brain congestion, petechial hemorrhage in the proventriculus, myocarditis
**Assiut**	35	—	Broilers, 23–31 days	5000–11000	30–50	Tracheal swab (14); Cloacal swab (10); Pancreas (6); Lung (28); Trachea (19); Kidney (12); Brain (11)	Inactivated H5N1 vaccine	4	—
**El-Minya**	25	—	Broilers, 25–35 days	5000–10000	27–45	—	Inactivated H5N1 vaccine + some unvaccinated	4	—

Tracheal and cloacal swabs, as well as tissue samples, were collected from freshly dead birds showing severe respiratory, digestive, and neurological signs, including dyspnea, nasal discharge, facial edema, cyanotic combs, greenish diarrhea, tremors, and head tilts. Postmortem findings included congested intestines, pulmonary edema, petechial hemorrhage in the proventriculus, and brain congestion.

Most examined birds had previously received commercial avian influenza (AIV) vaccines. The collected specimens were pooled (brain, pancreas, kidney, lung, and trachea) from each flock, homogenized in sterile phosphate-buffered saline (PBS; pH 7.4) supplemented with 10% antibiotic solution, and clarified by centrifugation at 1,107 × *g* for 15 min after overnight incubation at 4°C. The resulting supernatants were stored at −80°C until further analysis by virus isolation and polymerase chain reaction (PCR).

### Virus isolation in embryonated chicken eggs

A 0.2 mL aliquot of each processed supernatant was inoculated into the allantoic cavity of 9–11-day-old specific pathogen-free embryonated chicken eggs (SPF-ECE) obtained from Nile SPF, Koom Oshiem, Fayoum, Egypt, following the method of World Organization of Animal Health (WOAH) [33]. The eggs were incubated at 37°C for 4 days, with daily candling to monitor embryo viability. After overnight chilling, the allantoic fluids were harvested and screened for hemagglutination activity.

### Hemagglutination and HI assays

The HA assay was performed using 1% freshly prepared chicken red blood cells. HA-positive samples were preserved at −80°C for molecular characterization.

For serological confirmation, HI tests were performed using monospecific antisera against the AIV-H5N1 subtype (clone 11C5; Immune-Tech, Canada, Cat. No. IT-002-AIV-H5-EGY-11C5), derived from the reference strain A/chicken/Egypt/S3280D/2011 (H5N1). Additional antisera against H5N8 and NDV were used to verify antigenic purity [34]. The antisera were pre-treated with receptor-destroying enzyme II, heat-inactivated at 56°C for 30 min, and diluted 1:10 in PBS with 0.5% chicken erythrocytes. Results were interpreted as the reciprocal of the highest dilution completely HI, with a positivity cut-off of ≥1:16 (≥4 log2) according to WOAH guidelines [34].

### RNA extraction and real-time RT-PCR

RNA was extracted from HA-positive allantoic fluids using the QIAamp viral RNA mini kit (Qiagen, Hilden, Germany) according to the manufacturer’s instructions. Extracted RNA served as the template for one-step RT-PCR and real-time RT-qPCR assays targeting the matrix (*M*) gene of influenza A viruses [35]. Positive samples were further screened for H5, H6, H9, N1, N2, and N8 subtypes using the Verso 1-step real-time PCR kit (Thermo Fisher Scientific, Waltham, MA, USA; Cat. No. AB4101A) and primer sets as listed in Table 2 [36–38].

**Table 2:** Consensus primer and probe sets used for virus identification, subtyping, and sequencing in collected samples.

ID	Primer and probe sequences	Reference
*AIV-M*-gene	sep1: AGATGAGTCTTCTAACCGAGGTCG sep2: TGCAAAAACATCTTCAAGTCTCTG	[35]
	sep-probe: FAM-TCAGGCCCCCTCAAAGCCGA-TAMRA	
AIV-H5 subtype	H5LH1: ACATATGACTACCCACARTATTCAG H5RH1: AGACCAGCTAYCATGATTGC	[36]
	H5PRO: FAM-TCWACAGTGGCGAGTTCCCTAGCA-TAMRA	
AIV-H6 subtype	IAV-H6-1666F: CTTGGTGTGTATCAAATYCTTGC IAV-H6-1776R: CATTGARCCATTTGARCACATCCA	[38]
	IAV-H6-1693: FAM-TATAGTACGGTATCGAGCAGYCT-MGB	
AIV-H9 subtype	For: GGAAGAATTAATTATTATTGGTCGGTAC Rev: GCCACCTTTTTCAGTCTGACATT	[37]
	H9 probe: FAM-AACCAGGCCAGACATTGCGAGTAAGATCC-TAMRA	
AIV-N1 subtype	N1 forward: TAYAACTCAAGGTTTGAGTCTGTYGCTTG N1 reverse: ATGTTRTTCCTCCAACTCTTGATRGTGTC	[38]
	N1-Probe: FAM-TCAGCRAGTGCYTGCCATGATGGCA-TAMRA	
AIV-N2 subtype	FN2: TGGACAGGGAACAACACTAAA C RN2: ACAAGCCTCCCATCGTAAAT	[38]
	N2-Probe: TXRED-CAAATGAAATGGAACACCCAACTCAT-BHQ23	
AIV-N8 subtype	N8-1296F: TCCATGYTTTGGGTTGARATGAT N8 1423R: GCTCCATCRTGCCAYGACCA	[38]
	N8 A354-Probe: FAM-TCHAGYAGCTCCATTGTRATGTGTGGAGT-TAMRA	
IBV	AIBV-fr: ATGCTCAACCTTGTCCCTAGCA AIBV-as: TCAAACTGCGGATCATCACGT	[41]
	AIBV-TM: FAM-TTGGAAGTAGAGTGACGCCCAAACTTCA-TAMRA	
NDV	F +4839: TCCGGAGGATACAAGGGTCT F -4939: AGCTGTTGCAACCCCAAG	[39]
	F + 4894: FAM-AAGCGTTTCTGTCTCCTTCCTCCA-TAMRA	
ILTV	ILTVgCU771: CCTTGCGTTTGAATTTTTCTGT ILTVgCL873: TTCGTGGGTTAGAGGTCTGT	[46]
	ILTVprobe817: FAM-CAGCTCGGTGACCCCATTCTA-MGBNFQ	
IBDV	F/AUS GU: TCACCGTCCTCAGCTTACCCACATC R/AUS	[40]

### Screening for co-infection

All AIV-positive samples were concurrently tested for co-infection with other major avian respiratory pathogens, including NDV, IBV, ILTV, and IBDV, using established RT-qPCR protocols. The primer and probe sequences are detailed in Table 2 [39–42].

### Partial amplification and sequencing of the HA Gene

Partial amplification of the HA gene was performed using the EasyScript one-step RT-PCR kit (TransGen Biotech, Beijing, China) on an Applied Biosystems ProFlex PCR System (Waltham, Massachusetts, USA). Specific primers were used:

- KH1-Forward: *CCTCCAGARTATGCMTAYAAAATTGTC*
- KH3-Reverse: *TACCAACCGTCTACCATKCCYTG* [43]

The amplified 400 bp PCR products were visualized by agarose gel electrophoresis, purified using the QIAquick Gel Extraction Kit (Qiagen, Hilden, Germany), and used for sequencing.

### Phylogenetic and bioinformatics analysis

Purified PCR products were sequenced using the BigDye terminator v3.1 cycle sequencing kit (Applied Biosystems, USA) and analyzed on an Applied Biosystem (ABI) 3500×L Genetic Analyzer, Thermo Fisher Scientific Inc., Waltham, Massachusetts, USA. The obtained nucleotide sequences were deposited in GenBank under accession numbers PV017774–PV017775. Sequence alignment and phylogenetic analysis were performed using Clustal W, molecular evolutionary genetics analysis version 7.0 (MEGA 7.0), and BioEdit software packages [44, 45]. Phylogenetic trees were constructed using the maximum-likelihood method with 1,000 bootstrap replicates and the general time reversible model to determine genetic relationships with global reference strains and vaccine lineages.

## RESULTS

### Clinical and gross findings

Out of the 25 broiler flocks investigated, AIV was detected in four farms, yielding an overall prevalence rate of 16% (Table 1). Clinically, the infected birds from the New Valley, Assiut, and El-Minya governorates exhibited respiratory, digestive, and neurological manifestations. The most consistent respiratory signs included nasal and ocular discharge, coughing, dyspnea, and rales, often accompanied by facial swelling and cyanosis of the comb and wattles. Digestive involvement was evident through greenish diarrhea and reduced feed intake, whereas neurological signs such as paresis, tremors, opisthotonosis, and head tilt were frequently observed.

Signs of systemic septicemia were also apparent, including subcutaneous hemorrhages of the shank, comb cyanosis, and generalized weakness, resulting in high morbidity (~80%) and mortality rates ranging from 25% to 50%.

**Figure 2:**
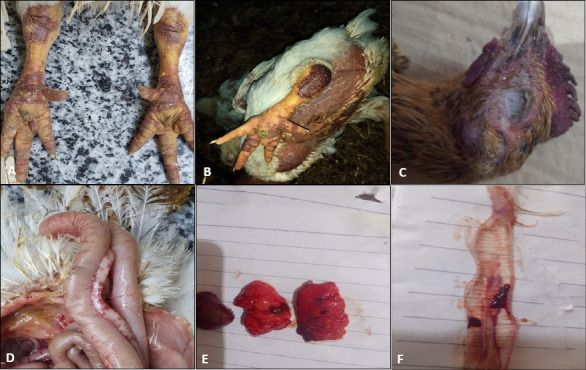
The characteristic clinical signs and postmortem lesions of Avian influenza virus observed on live or dead broilers (randomly selected examples) during sampling. (A) Ecchymotic hemorrhage on the shank and leg. (B) Postmortem examination of freshly dead broilers showing severe muscular congestion. (C) Cyanosis in the comb and wattles, and periorbital edema. (D) Hemorrhages on the pancreas. (E) Enlarged congested spleen and lung. (F) Tracheitis with pneumonia.

Postmortem examinations revealed typical HPAI lesions, including facial edema, pulmonary congestion, petechial hemorrhage in the proventriculus, congested intestines, and multifocal necrosis in the liver and pancreas. Additional findings included tracheitis, pneumonia, and subepicardial hemorrhage (Figure 2), confirming severe systemic infection consistent with HPAI.

### Virus isolation and molecular identification

**Figure 3:**
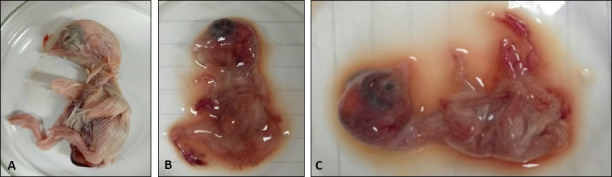
Specific-pathogen-free embryonated chicken eggs lesions of Avian influenza virus (AIV)-infected embryos. (A) Normal embryo (control) on the left side. (B and C) AIV-infected embryos showing severe acute hemorrhages and congestion (randomly selected examples). These embryos died at 48 h and 72 h.

Five H5N1-positive specimens were successfully isolated using SPF-ECE. The isolates produced hemagglutination titers of 7–8 log2, confirming active viral replication (Figure 3). The allantoic fluids exhibited HI titers ranging from 5 to 7 log_2_ when tested with H5N1-specific antiserum, but no inhibition with antisera against H5N8 or NDV, confirming antigenic purity.

**Figure 4:**
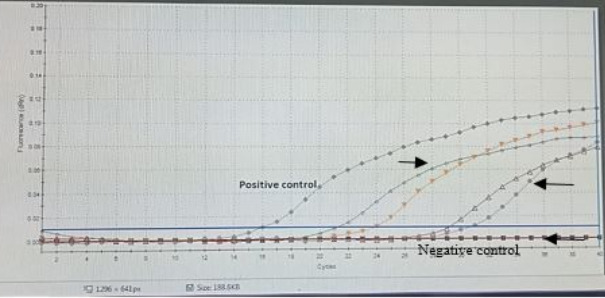
Avian influenza virus reverse-transcription quantitative polymerase chain reaction (RT-qPCR) assay amplification plot showing four positive H5 subtype isolates (between two arrows) generated by the Stratagene MX3005P software (Agilent Technologies, Inc., California, USA) with positive and negative controls. The cycle threshold cut-off values for the four RT-qPCR samples were 19.27, 21.14, 26.33, and 28.15, respectively.

**Figure 5:**
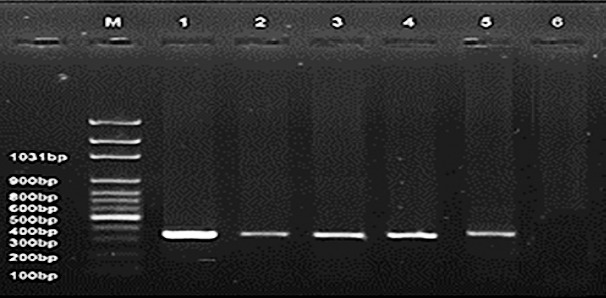
Identification of H5 subtyping by reverse-transcription quantitative polymerase chain reaction for avian influenza virus-positive isolates using H5 primers. The 400-bp amplicon indicates the four H5 subtypes. Lanes 1–4 are positive samples, and lanes 5 and 6 are positive and negative controls, respectively. Lane M = Represents a 100-bp ladder as a size standard.

Using real-time RT-PCR targeting the matrix *(M)* gene, four samples were confirmed positive for AIV-H5N1 subtype (Figure 4). The remaining samples tested negative for other viral pathogens, including NDV, IBV, ILT, and IBDV (Figure 5), suggesting a single viral infection without co-infection interference.

### Sequencing and phylogenetic characterization of the H5N1 strains

**Figure 6:**
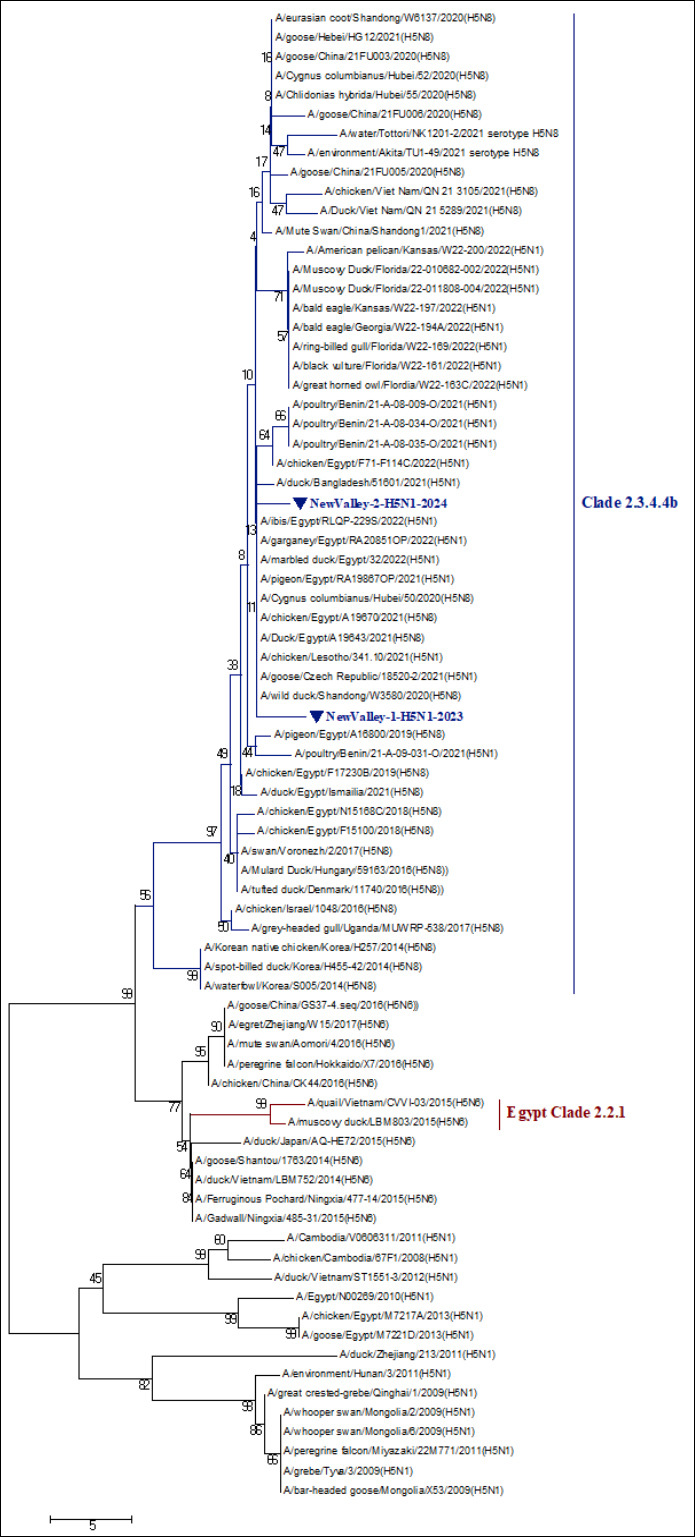
Collective phylogenetic tree of the nucleotide sequence alignments of HA gene segments (partial sequencing) of various avian influenza virus (AIV) H5 subtype strains with other reference strains and Egyptian strains deposited in GenBank. AIV H5 subtype: AIV H5 subtype. The phylogenetic analysis of the HA gene revealed that two Egyptian highly pathogenic avian influenza virus-H5N1 isolates (indicated by blue triangles) were located in clade 2.3.4.4b. The tree was constructed using the maximum-likelihood method with 1000 bootstrap replicates in Molecular Evolutionary Genetics Analysis version 7.0, under the general likelihood reversible substitution model, and a bootstrap value of 1,000 replicates.

**Figure 7:**
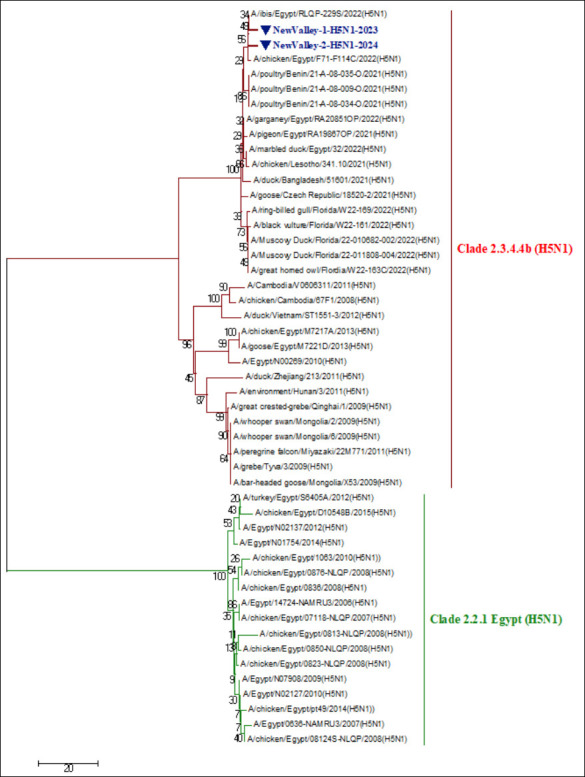
Detailed phylogenetic tree of the highly pathogenic avian influenza virus-H5N1 strains within clade 2.3.4.4 b based on partial HA gene segment sequencing comparable to other referential strains and Egyptian strains deposited in GenBank. The phylogenetic analysis of the HA gene confirmed that two Egyptian HPAI-H5N1 isolates (indicated by blue triangles) were in clade 2.3.4.4b. The tree was constructed using the maximum-likelihood method with 1000 bootstrap replicates using molecular evolutionary genetics analysis version 7.0 software under the general likelihood reversible substitution model and a bootstrap value of 1000 repeats.

Partial sequencing of the *HA* gene from two representative isolates, New Valley-1-H5N1-2023 and New Valley-2-H5N1-2024, confirmed their classification as HPAI-H5N1 belonging to clade 2.3.4.4b. Both isolates were submitted to GenBank under accession numbers PV017774–PV017775 (Figures 6 and 7).

Molecular analysis revealed a multibasic cleavage site motif (PLREKRRKR/GLF; residues 321–332) characteristic of highly pathogenic strains. The HA proteins exhibited amino acid substitutions R72S in the receptor-binding region and A83D, T140A in antigenic site A, indicating possible antigenic drift within circulating Egyptian field strains.

### Phylogenetic relatedness to global and local strains

Phylogenetic analysis of the *HA* gene demonstrated that both isolates clustered tightly within the HPAI-H5N1 clade 2.3.4.4b group, showing close genetic relatedness to strains circulating in Bangladesh, China, Lesotho, and the Czech Republic during 2021–2022 (Figures 6 and 7). Likewise, the isolates shared 96%–99% nucleotide and amino acid identity with recent Egyptian H5N1 isolates, including A/ibis/Egypt/RLQP-229S/2022 (H5N1), A/garganey/Egypt/RA20851OP/2022 (H5N1), A/chicken/Egypt/F71-F114C/2022 (H5N1), and A/marbledduck/ Egypt/32/2022 (H5N1) (Table 3).

**Table 3:** Amino acid and nucleotide identities and divergence of partially sequenced AIV isolates compared with other selected strains, including vaccinal strains. The table presents a comparative alignment of the H5N1, showing that the nucleotide and amino acid identity percentages of our two Egyptian highly pathogenic avian influenza virus-H5N1 isolates range from 72% to 99%, comparable to other reference strains. The blue color indicates commercial AIV vaccines in Egypt.

ID / Sequence	A	B	C	D	E	F	G	H	I	J	K	L	M	N	O	P	Q	R	S	T	U	V	W
A	ID	99	99	99	98	83	84	84	84	97	98	98	98	98	98	83	84	84	73	84	83	98	99
B	99	ID	98	100	99	84	84	85	85	98	99	98	98	99	99	83	84	84	74	85	84	97	98
C	99	98	ID	98	98	83	84	84	84	97	97	97	97	98	97	83	84	84	72	84	83	97	98
D	99	100	98	ID	99	84	84	84	84	97	98	98	98	99	98	83	84	84	74	85	83	97	98
E	98	99	98	99	ID	84	84	84	84	97	98	98	98	98	98	83	84	84	74	85	83	96	98
F	83	84	83	84	84	ID	89	90	90	85	84	85	84	84	83	89	88	88	74	91	88	81	82
G	84	84	84	84	84	89	ID	97	97	86	84	85	83	84	84	95	91	95	76	98	91	82	84
H	84	85	84	84	84	90	97	ID	100	86	84	85	84	84	84	98	91	97	75	100	91	82	84
I	84	85	84	84	84	90	97	100	ID	86	84	85	84	84	84	98	91	97	75	100	91	82	84
J	97	98	97	97	97	85	86	86	86	ID	98	99	97	97	98	85	85	85	74	86	86	95	96
K	98	99	97	98	98	84	84	84	84	98	ID	98	100	98	98	83	84	84	74	84	84	96	97
L	98	98	97	98	98	85	85	85	85	99	98	ID	98	98	98	84	85	85	74	86	85	96	97
M	98	98	97	98	98	84	83	84	84	97	100	98	ID	98	82	84	83	74	84	83	96	97	97
N	98	99	98	99	98	84	84	84	84	97	98	98	98	ID	98	83	84	84	74	85	83	96	98
O	98	99	97	98	98	83	84	84	84	98	98	98	98	98	ID	84	84	85	74	84	84	96	97
P	83	83	83	83	83	89	95	98	98	85	83	84	82	83	84	ID	90	98	76	98	90	81	82
Q	84	84	84	84	84	88	91	91	91	85	84	85	84	84	84	90	ID	90	72	91	96	82	83
R	84	84	84	84	84	88	95	97	97	85	84	85	83	84	85	98	90	ID	75	97	89	82	83
S	73	74	72	74	74	74	76	75	75	74	74	74	74	74	74	76	72	75	ID	76	74	72	74
T	84	85	84	85	85	91	98	100	100	86	84	86	84	85	84	98	91	97	76	ID	91	82	84
U	83	84	83	83	83	88	91	91	91	86	84	85	83	83	84	90	96	89	74	91	ID	81	83
V	98	97	97	97	96	81	82	82	82	95	96	96	96	96	96	81	82	82	72	82	81	ID	97
W	99	98	98	98	98	82	84	84	84	96	97	97	97	98	97	82	83	83	74	84	83	97	ID

A = A/ibis/Egypt/RLQP-229S/2022(H5N1),

B = A/garganey/Egypt/RA20851OP/2022(H5N1),

C = A/chicken/Egypt/F71-F114C/2022(H5N1),

D = A/marbled duck/Egypt/32/2022(H5N1),

E = A/pigeon/Egypt/RA19867OP/2021(H5N1),

F = A/duck/Zhejiang/213/2011(H5N1),

G = A/Egypt/N00269/2010(H5N1),

H = A/chicken/Egypt/M7217A/2013(H5N1),

I = A/goose/Egypt/M7221D/2013(H5N1),

J = A/chicken/Israel/1048/2016(H5N8),

K = A/chicken/Egypt/A19670/2021(H5N8),

L = A/chicken/Egypt/F15100/2018(H5N8),

M = A/duck/Egypt/A19643/2021(H5N8),

N = A/goose/Czech Republic/18520-2/2021(H5N1),

O = A/duck/Egypt/Ismailia/2021(H5N8),

P = A/chicken/Egypt/D10552B/2015(H5N1),

Q = A/chicken/Egypt/Q1995D/2010(H5N1),

R = A/chicken/Egypt/RG-173CAL/2017(H5N1),

S = A/chicken/Mexico/232/94(H5N2),

T = A/duck/Egypt/M2583D/2010(H5N1),

U = A/chicken/Egypt/18-H/2009(H5N1),

V = New Valley-1-H5N1-2023,

W = New Valley-2-H5N1-2024

The New Valley isolates also demonstrated high sequence homology (95%–97%) with Egyptian and Israeli H5N8 strains, such as A/chicken/Israel/1048/2016 (H5N8) and A/duck/Egypt/A19643/2021 (H5N8), suggesting shared ancestry and inter-clade reassortment events within clade 2.3.4.4b.

In contrast, marked genetic divergence (72%–84%) was observed when compared with commercial vaccine strains currently used in Egypt, including MEFLUVAC [Kemin Industries, Inc.] (Clade 2.2.1.2), EgyFlu [Nagy Awad Group] (Clade 2.2.1.1), and Nobilis H5N2 [MSD Animal Health]. These low identity percentages at both nucleotide and amino acid levels underscore the antigenic mismatch between circulating field strains and existing vaccines.

Comparative sequence analysis revealed 97% nucleotide and amino acid similarity between the two newly sequenced isolates themselves, confirming their close genetic relationship and probable shared origin. Collectively, these results demonstrate that Egyptian H5N1 strains in Upper Egypt are phylogenetically aligned with the global clade 2.3.4.4b lineage, showing extensive regional and intercontinental connectivity.

### Summary of genetic identity patterns

Overall, the New Valley-1-H5N1-2023 and New Valley-2-H5N1-2024 isolates:

1)Clustered within clade 2.3.4.4b alongside Eurasian and African H5N1 strains (2021–2022).
2)Shared 96%–99% similarity with recent Egyptian H5N1 field strains.
3)Displayed 95%–97% relatedness with Egyptian and Israeli H5N8 viruses, indicating cross-clade evolutionary linkage.
4)Showed only 72%–84% homology with commercial vaccine strains, suggesting a potential vaccine failure risk.

These findings highlight the genetic evolution and antigenic drift of circulating H5N1 viruses in Upper Egypt and the urgent need for vaccine seed strain updates to improve protection against current field isolates.

## DISCUSSION

### Global evolution and spread of HPAI-H5N1

The HPAI H5N1 virus was first identified in 1996 in domestic poultry in Guangdong, China, and has since spread worldwide through migratory waterfowl, causing severe outbreaks in poultry and sporadic human infections [47, 48]. Continuous global circulation has promoted the accumulation of adaptive, non-silent mutations in surface glycoproteins (HA and NA), a process known as antigenic drift, which enables viral escape from host immunity.

To date, AIVs have been classified into nine major clades with multiple subclades. Among these, the H5Nx viruses within clade 2.3.4.4 (subgroups a–h) have shown the highest frequency of reassortment and intercontinental spread [23]. In recent years, clade 2.3.4.4b H5N1 variants, first recognized in 2020, have become the dominant global lineage, causing devastating outbreaks in wild birds and poultry, and occasionally in humans [23, 25].

### Epidemiological situation in Egypt

In Egypt, many viral diseases have been reported, causing multiple outbreaks [49–52]. Egypt has been endemic for AIV infections since early 2006, following the introduction of H5N1 clade 2.2 viruses in 2005 [53, 54].

Despite extensive vaccination campaigns, successive outbreaks have occurred, driven by continuous viral evolution. In late 2021, the H5N1 subtype within clade 2.3.4.4b was introduced into Egypt, likely through migratory birds along the Black Sea–Mediterranean flyway, and is now co-circulating with H5N8 subtypes of the same clade [55]. The present study represents one of the first molecular investigations of HPAI-H5N1 clade 2.3.4.4b in broiler flocks from Upper Egypt (2023–2025).

### Field observations and clinical correlations

The detection of AIV in 4 of 25 flocks (16%) reflects continued viral activity in commercial broilers of Upper Egypt. The affected birds exhibited classical respiratory, digestive, and neurological signs, including dyspnea, nasal discharge, facial swelling, greenish diarrhea, and tremors, accompanied by 25%–50% mortality and ~80% morbidity. These manifestations are consistent with previously documented H5N1 outbreaks [55–58].

The occurrence of infection in previously vaccinated flocks highlights vaccine failure, likely due to improper administration, inadequate antigenic mass, poor cold-chain maintenance, host factors (age, immune suppression), or antigenic drift reducing cross-protection [59, 60]. Following SPF-ECE inoculation, embryos showed acute hemorrhagic lesions and congestion, similar to earlier reports [61, 62].

### Molecular detection and absence of co-infection

RT-qPCR remains the most rapid and reliable diagnostic tool for identifying reassortant and emerging AIV strains [36]. In this study, only four samples tested positive for the H5N1 subtype, while all were negative for NDV, IBV, ILT, and IBDV, excluding mixed viral infections. This pattern matches previous Egyptian surveillance findings [55, 60].

### Phylogenetic and evolutionary insights

Molecular sequencing of two representative isolates, New Valley-1-H5N1-2023 and New Valley-2-H5N1-2024, confirmed their identity as HPAI-H5N1 (clade 2.3.4.4b). The isolates shared 97% nucleotide and amino acid similarity, clustering closely with contemporary Eurasian and African strains (2021–2022), including those from China, Bangladesh, Lesotho, the Czech Republic, and Benin. This finding indicates that the Upper Egypt isolates likely originated via migratory bird movements along major flyways, supporting the concept of avian-mediated transcontinental dissemination of AIVs.

Such phylogenetic alignment underscores the need for integrated surveillance systems that combine molecular monitoring with ecological tracking of migratory species, enabling early detection and risk mitigation.

### Molecular mutations and antigenic drift

The two isolates exhibited key amino acid substitutions (R72S, A83D, T140A) within the HA immunogenic regions.

- A83D and T140A occur within antigenic site A, potentially altering epitope exposure and facilitating immune escape.
- R72S affects the receptor-binding region, possibly modifying host-receptor affinity without drastically altering antigenicity.

These mutations signal ongoing antigenic remodeling, a hallmark of viral adaptation under immunological pressure [61–66]. The HA cleavage motif PLREKRRKR/GLF, conserved among Egyptian isolates, reaffirms the highly pathogenic phenotype [67, 68].

Similar mutations have been described in earlier Egyptian and Asian H5N1 lineages, highlighting the virus’s dynamic molecular evolution and its tendency to generate drift variants under vaccine-driven selection.

### Comparative genetic relationships

Nevertheless, Abd El-Hamid *et al*. [69] reported five isolates HPAI-H5N1 viruses belonging to the dominant subtype 2.2.1.2, in Upper Egypt governorates (Assiut, El-Minya, and New Valley) in 2017 based on partial HA sequencing. In addition, Arafa *et al*. [70] identified H5N1 strains of clade 2.2.1.2 in six Upper Egypt governorates during the year 2014-2015. The New Valley isolates showed 96%–99% identity with Egyptian H5N1 clade 2.3.4.4b strains detected during 2021–2022 and 95%–97% homology with H5N8 clade 2.3.4.4b viruses circulating between 2018 and 2021. This high similarity suggests common ancestry or reassortment events between H5N1 and H5N8 lineages. In contrast, nucleotide and amino acid identity with commercial vaccine strains, MEFLUVAC [Kemin Industries, Inc.] (clade 2.2.1.2), EgyFlu [Nagy Awad Group] (clade 2.2.1.1), and Nobilis H5N2 [MSD Animal Health], was only 72%–84%, confirming a substantial antigenic mismatch.

Comparable observations were reported by Setta *et al*. [61], who found vaccine–field strain identities ranging from 75.8% to 90.7%, which explains the reduced protective efficacy. These data emphasize that the existing vaccines are poorly matched to circulating clade 2.3.4.4b viruses, leading to continued outbreaks despite vaccination.

### Implications for control and vaccination strategies

The demonstrated genetic divergence between current field isolates and commercial vaccine seeds underscores the urgent need to update Egypt’s avian influenza vaccination program. Developing locally tailored vaccines incorporating contemporary H5N1 clade 2.3.4.4b antigens would enhance protection and mitigate economic losses. Moreover, strengthening biosecurity, maintaining cold chains, and increasing vaccination coverage across poultry farms remains essential.

Integration of One Health approaches across the veterinary, wildlife, and public health sectors will further aid in preventing zoonotic transmission and advancing SDG 3 (Good Health and Well-Being).

This study provides the first molecular evidence of HPAI-H5N1 clade 2.3.4.4b circulation in broiler flocks of Upper Egypt. The isolates exhibit close genetic relationships with Eurasian field strains and low similarity to current vaccine strains, indicating active viral evolution and potential vaccine mismatch. Continuous genetic surveillance, vaccine updates, and regional cooperation are essential for controlling the spread of HPAI and safeguarding both poultry health and public health within a *One Health* framework.

## CONCLUSION

This study provides the first molecular evidence of HPAI H5N1 clade 2.3.4.4b circulating among broiler flocks in Upper Egypt during 2023–2025. Of the 25 examined flocks, 4 were confirmed positive, yielding a prevalence of 16% and mortality rates ranging from 25% to 50%. The infected birds exhibited typical respiratory, digestive, and neurological signs, as well as postmortem lesions characteristic of HPAI. Molecular detection through RT-qPCR and hemagglutination/HI assays confirmed the presence of the H5N1 subtype, while sequencing of the *HA* gene identified two representative isolates (New Valley-1-H5N1-2023 and New Valley-2-H5N1-2024) within clade 2.3.4.4b, sharing 96%–99% nucleotide and amino acid identity with recent Egyptian and Eurasian field strains.

The observed low genetic similarity (72%–84%) with commercial vaccine strains (MEFLUVAC [Kemin Industries, Inc.], EgyFlu [Nagy Awad Group], and Nobilis H5N2 [MSD Animal Health]) indicates a significant antigenic mismatch and is likely the cause of vaccine failure in vaccinated flocks. These findings necessitate the urgent updating of vaccine seed strains and the enhancement of surveillance programs, particularly in Upper Egypt, which lies along major migratory bird flyways. Incorporating locally circulating clade 2.3.4.4b antigens into new vaccine formulations will improve protection, reduce mortality, and limit viral shedding.

The strength of this work lies in its integration of clinical, molecular, and phylogenetic evidence, revealing new genetic insights from an understudied region. However, the study was limited by partial *HA* gene sequencing, which precludes full-genome evolutionary interpretation and excludes analysis of NA and polymerase genes that might contribute to virulence and host adaptation. Future research should involve whole-genome sequencing, antigenic cartography, and experimental vaccine-matching studies to guide national control policies. Continued genomic monitoring and wild bird surveillance will be crucial for mapping viral movement, predicting reassortment events, and mitigating cross-species transmission risks.

Collectively, this study underscores the ongoing evolution of H5N1 clade 2.3.4.4b in Egypt, the urgent need for updated vaccines, and the importance of One Health-based surveillance integrating animal, wildlife, and human health sectors to achieve sustainable disease control and pandemic preparedness.

## DATA AVAILABILITY

The datasets generated and analyzed during the current study are available at https://blast.ncbi.nlm.nih. gov/Blast.cgi or accession number to datasets: [PV017774–PV017775].

## AUTHORS’ CONTRIBUTIONS

EAES, MSA, and MAEM: Designed the study. MKM, MHM, and MSA: Performed the collection and processing of the samples. EAES: Performed the sequencing, phylogenetic analysis, computational analysis of the data, and performed the laboratory virological experiments. EAES and IMD: Wrote and reviewed the manuscript. All authors have read and approved the final version of the manuscript.
